# *Salicornia bigelovii*, *S. brachiata* and *S. herbacea*: Their Nutritional Characteristics and an Evaluation of Their Potential as Salt Substitutes

**DOI:** 10.3390/foods11213402

**Published:** 2022-10-28

**Authors:** Hani A. Alfheeaid, Dele Raheem, Faiyaz Ahmed, Fahad S. Alhodieb, Zayed D. Alsharari, Jwaher Haji Alhaji, Mona N. BinMowyna, Ariana Saraiva, António Raposo

**Affiliations:** 1 Department of Food Science and Human Nutrition, College of Agriculture and Veterinary Medicine, Qassim University, Buraydah 51452, Saudi Arabia; 2 Northern Institute for Environmental and Minority Law (NIEM), Arctic Centre, University of Lapland, 96101 Rovaniemi, Finland; 3 Department of Clinical Nutrition, College of Applied Health Sciences in Ar Rass, Qassim University, Ar Rass 51921, Saudi Arabia; 4 Department of Clinical Nutrition, Faculty of Applied Medical Sciences, University of Tabuk, Tabuk 71491, Saudi Arabia; 5 Department of Health Sciences, College of Applied Studies and Community Service, King Saud University, Riyadh 4545, Saudi Arabia; 6 College of Applied Medical Sciences, Shaqra University, Shaqra 11961, Saudi Arabia; 7 Department of Animal Pathology and Production, Bromatology and Food Technology, Faculty of Veterinary, Universidad de Las Palmas de Gran Canaria, Trasmontaña s/n, 35413 Arucas, Spain; 8 CBIOS (Research Center for Biosciences and Health Technologies), Universidade Lusófona de Humanidades e Tecnologias, Campo Grande 376, 1749-024 Lisboa, Portugal

**Keywords:** *Salicornia* spp., *S. bigelovii*, *S. brachiata*, *S. herbacea*, salt substitute, nutrition, food industry, health, antioxidant, anti-inflammatory

## Abstract

Excessive sodium (salt) intake in our diet is a main contributor to hypertension and a major risk factor for cardiovascular illnesses. As a result, research has made great efforts to develop salt alternatives, and *Salicornia* spp. offers a very high potential in the food industry for its promising functional characteristics. This review focuses on the nutritional profile, health effects and commercial potential of three specific species of the *Salicornia* genus: *S. bigelovii*, *S. brachiata* and *S. herbacea*. It also addresses the methods that are used to produce them as salt substitutes. Owing to the antinutritional and anti-inflammatory effects of its bioactive compounds, *Salicornia* spp. can serve as an organic biological preservative in foods with better consumer appeal when compared with chemical preservatives that are common in the food industry. Overall, the commercial use of these underutilized species will help to improve food security.

## 1. Introduction

*Salicornia,* a genus of annual, rapidly growing euhalophytes, belongs to the Chenopodiaceae family. It has fleshy, articulated stems, scale-like leaves and flowers arranged in spike-like dense and thick thyrses [1,2]. *Salicornia* spp. often start their life cycles in spring as green plants, gradually become reddish-yellow in autumn and die in winter. In taxonomical terms, the *Salicornia* genus is complex with several species, aggregates and subspecies [3]. All around the world, *Salicornia* spp. naturally exist on saline inlands and in coastal locations, including saltpans and salt marshes. They feature among the most promising halophytes for commercial production because of their wide-ranging resistance to climate and salinity conditions [4,5]. *Salicornia* is eaten fresh, fermented, cooked, dehydrated or pickled (seasoning). Since ancient times, it has been extensively used in coastal regions of Asia, particularly as fermented food and seasoning. It is also employed in folk medicine to cure various ailments, including obesity, diabetes, constipation and cancer [6]. Consuming *Salicornia* is more recent and less common in North American and European markets and is quite appreciated in the gourmet market. Demand surpasses supply in certain nations, including the UK and Ireland, especially in winter when imports are required from other nations such as Portugal [5]. In addition to *haute cuisine*, traditional cuisine is starting to show an interest in using it as a salt alternative, but it presently comes in a powdered form. *Salicornia* is a promising novel functional component for its significant nutritional value, including its mineral richness and fiber content [6]. Additionally, research has demonstrated that *Salicornia* is an excellent source of bioactive substances (e.g., polysaccharides, phytosterols and phenolic compounds) for cosmetic and nutraceutical industries, which all make it a crucial component of goods marketed to rapidly expand natural and organic markets [7]. In addition to these uses, seeds are utilized for extracting high-quality edible oil, and leftover plants are employed as forage. Given its huge potential as a crop in saline soil, *Salicornia* is a very adaptable plant that possesses a range of applications and is now considered to be one of the “plants of the future” [5,7,8].

Between 0.18 and 0.23 g/day of sodium must be consumed daily to sustain essential biological functions [9]. However, in our modern culture, individuals often eat excessive sodium (salt), which has become an issue in Western nations and a problem in developing nations that have acquired a liking for intake a high-sodium diet [10]. According to Powles et al. [11], the average overall sodium in 2010 was 3.95 g/day, with regional intakes falling within the 2.18–5.51 g/day range (sub-Saharan Africa to Central Asia). This amount more than doubles the recommended World Health Organization’s (WHO) limit of 2 g sodium/day (this equals 5 g/d of salt intake) [12]. Additionally, 181 of 187 countries (99.2% of the adult population) report higher sodium consumption levels than WHO recommendations; in 119 of these countries (88.3% of the world’s adult population), sodium intake exceeds the recommended threshold value of 1 g/d. In 51 countries (44.8% of the world’ adult population), the estimated average sodium intake is more than two-fold the recommended level. Asians, followed by Europeans, are the populations with the highest sodium intakes worldwide [11]. According to recent data, studies on salt intake were conducted between January 2011 and September 2018, with findings ranging from 6.75 g/d (6.32–7.17) in Barbados [13] to 10.66 g/d (10.52–10.81) in Portugal [14]. Consuming too much sodium is associated with illnesses such as hypertension and cardiovascular diseases [15]. As a result, the creation of salt substitutes has been paid a lot of attention by researchers [16,17,18]. Indeed, numerous research works have been conducted recently to examine the potential of *Salicornia* spp. as a functional component for the food industry in light of its promising functional traits. These research works have led to novel products with enhanced qualities being developed, including a fiber-enriched fermented milk probiotic [19] and vinegar with antifatigue and antioxidant effects [20]. *Salicornia* spp. has also been discovered to be a fascinating potential salt alternative [6,16,21,22,23,24].

Based on these premises, this review aims to study in depth three particular species of the *Salicornia* genus (*S. bigelovii*, *S. brachiata*, *S. herbacea*) by focusing on: their commercial potentialities on various industries including the food market; the techniques to produce them as salt substitutes; and both their nutrition profile and health impacts.

## 2. Generalities of the Genus *Salicornia*

The family Amaranthaceae (formerly Chenopodiaceae) includes the genus *Salicornia*. This annual succulent plant has leafless stems and branches whose sessile flowers are frequently grouped in three-flower cymes per bract and aggregated in dense terminal spike-like thyrses [3]. Inbreeding appears to dominate reproductive biology in diploid species. However, out-crossing happens, especially in tetraploid species such as *S. bigelovii* [25]. The majority of species favor unshaded locations, have an erect or prostate growing style and range in height (10–60 cm) and degree of branching (depending on environmental and climate circumstances) [26]. *Salicornia* species may be found growing inside and near inland and coastal salt lakes, saltpans, salt marshes and mudflats across boreal, temperate and subtropical regions in the northern hemisphere. The length of time that *Salicornia* spends submerged, the amount of waterlogging and salinity levels fluctuate in the daytime and across seasons. *Salicornia* spp. have been discovered to have a large amount of physiological plasticity, which results in wide-ranging phenotypic diversity between populations in various environmental circumstances [27]. *Salicornia* spp. have evolved exceptional salt tolerance to deal with the demanding edaphic conditions present in salt marsh ecosystems, where salinities can almost double the concentration of seawater (1 M NaCl) [8,28,29]. This high salt tolerance depends on the compartmentalization of salts in vacuoles, together with the production of suitable solutes. This allows for osmotic adjustment while also preventing the harmful effects of Cl^−^ and Na^+^ in the cytosol [30]. Compatible solutes, including sucrose, proline and the glycine-betaine function, act as osmoprotective substances that preserve protein integrity and shield the cytosol from ion toxicity and free radicals and maintain osmotic pressure [31].

Although no consensus about the precise numbers of recognized species has been reached, the *Salicornia* genus has 25–30 species [3]. A complex taxonomy has resulted from the high physiological adaptability level combined with a very restricted leaf and flower morphology that only provides a few identifying features [3,32]. The names *Salicornia europaea* L. and *Salicornia herbacea* L. have been broadly used to refer to a variety of genotypes due to taxonomic definition difficulties, and many names have been given to the same species depending on the area [26,32]. *Salicornia* is also called samphire, sea asparagus, pickled seaweed, crow’s foot green, hamcho, glasswort or sea beans depending on the location [33]. Although genetically different forms have been established by analyzing ribosomal DNA polymorphism and external transcribed spacer (ETS) sequence data, these methods have not been adequate for morphologically identifying distinct species [25,34]. Seed and fruit characteristics have been acknowledged as being potentially helpful diagnostic features for identifying species [35].

Despite the taxonomic challenges posed by both morphological parallelism and phenotypic plasticity, certain identified species have drawn more attention than others [36].

## 3. *Salicornia bigelovii*

**Figure 1:**
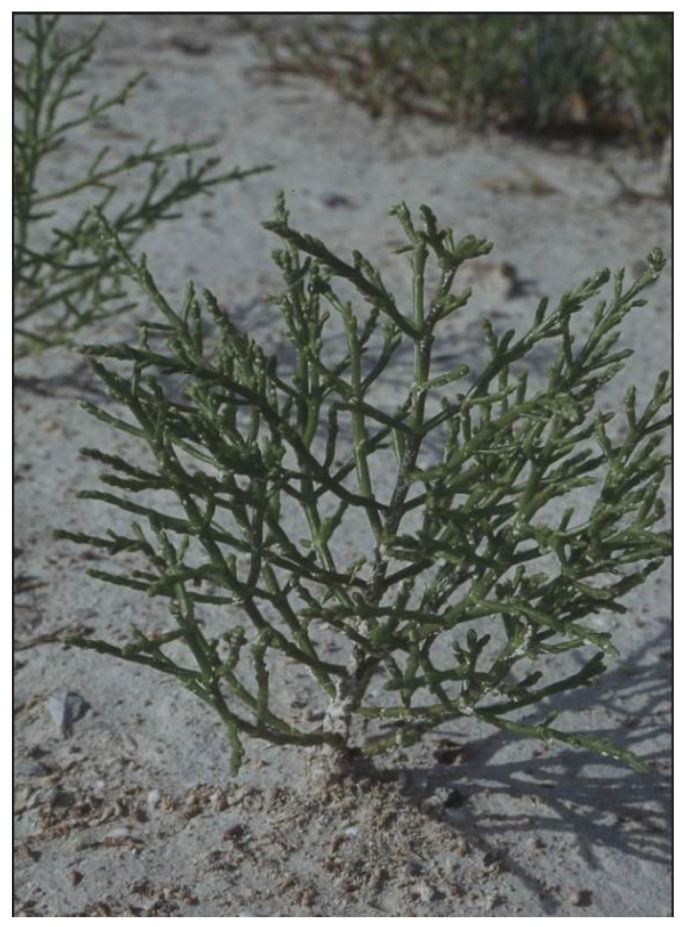
Photograph of *Salicornia bigelovii* in its natural habitat (Windsor Lake, CO, USA).

The dwarf glasswort *S. bigelovii* (Figure 1), which is a member of the *Salicornia* tetraploid branch native to North America, may be identified from other species by the tips of its bracts and leaves, which are acute and sharply mucronate [3]. Its growth habit is erect, and it can grow as tall as 50 cm in subtropical areas. It has been one of the most sought-after species in the endeavor to produce halophytes in coastal desert areas using seawater [29,37,38,39,40], and it can accumulate salt from up to 37% to 52% in dry mass [41]. The stem of this plant is succulent, erect and photosynthetic. We can find *S. bigelovii* in the coastal estuaries of the Mexican Sonora and Baja California States and in salt flats [42]. Several experts regard it as the most salt-tolerant vascular plant worldwide [43]. *S. bigelovii*, also known as samphire, is a pickled sea vegetable employed in both appetizer platters and salads. Given its oceanic flavor, raw samphire pairs well with fish and seafood. The halophyte’s leaves can be utilized as a fodder crop alternative to Rhodes grass [29] and alfalfa with livestock such as sheep and goats [44].

By using seawater for irrigation, *S. bigelovii* has the enormous potential for being exploited as an oilseed crop in coastal regions of wastelands and deserts [29]. On Mexico’s arid coastline, it has been assessed as an oilseed crop [45]. This halophyte may be grown as an oilseed crop in the sandy areas bordering the Gulf of California, the Indian Ocean, the Arabian Gulf and the Red Sea [37]. It is the most promising future oilseed halophyte crop for its seed’s high oil contents (30%) and low salt concentration (less than 3%) [37]. Its oil is acknowledged as good quality because it has high linoleic acid (75%) and linolenic acid (2%) levels, which are, respectively, two important omega-3 fatty acids for human nutrition and help to fight coronary heart disease [46,47]. Its meal has a high protein content (42–45%), which renders it suitable for use as animal feed [29]. Bearing in mind the significance of *S. bigelovii*, a breeding program has been launched in the USA and Eritrea thanks to its enhancement [48]. *S. bigelovii* has been cultivated on several lines to be used as vegetables, fodder and oilseeds by the Saudi Arabian BEHAR (the Arabian Saline Water Technology Company Ltd., Jubail, Saudi Arabia), which has been researching several halophyte plant species. *S. bigelovii* is characterized by a high protein content with high concentration of amino acids (Glu, Asp, Cyst and Gly) in both its shoots and seeds, and high unsaturated fatty acids were also reported [49]. The authors also acknowledged its role as a valuable source of minerals, amino acids and antioxidants, which makes it valuable as a food ingredient.

## 4. *Salicornia brachiata*

**Figure 2:**
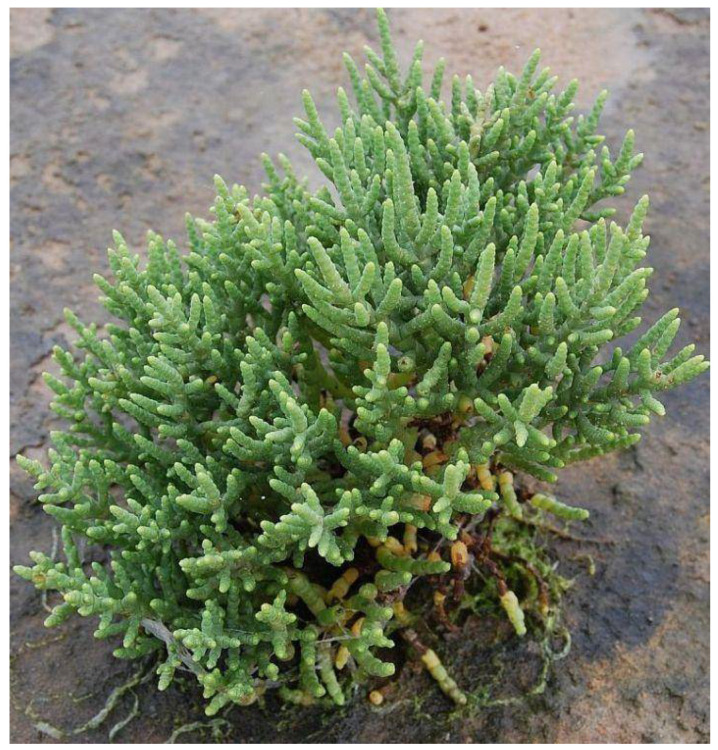
Photograph of *Salicornia brachiata* in its natural habitat (Gujarat, India).

With terminal fruit-bearing spikes and jointed, green succulent stems, *S. brachiata* (Figure 2) is one of some annual halophytes that can withstand high salt concentrations and grows without leaves [50]. According to Joshi et al. [51] and Glenn et al. [47], this plant is capable of thriving in salt marshes and even needs NaCl for in vitro regeneration. It also collects 30–40% NaCl in dry weight [52]. The plant is grown in high-salinity locations, commonly in the Gujarat coastal marshes of India, and is seen as a potential substitute seawater crop [52,53], making it an interesting option for animal feed. Given its protein-rich shoots and seeds, which are employed as salad greens, this plant provides nutritional benefits [54]. This extreme halophyte is a prospective contender with a wide range of applications given its potential for acting as a naturally adapted higher plant model for abiotic stress-responsive gene resources [55,56].

*S. brachiata* has been tested for its antiviral activities and is acknowledged as a traditional medicine to treat hepatitis [57]. There are reports about the presence of bioactive components, minerals, amino acids, polyphenols, proteins, reducing sugars and pigments with antioxidant qualities such as betacyanin and betaxanthin [58,59].

## 5. *Salicornia herbacea*

**Figure 3:**
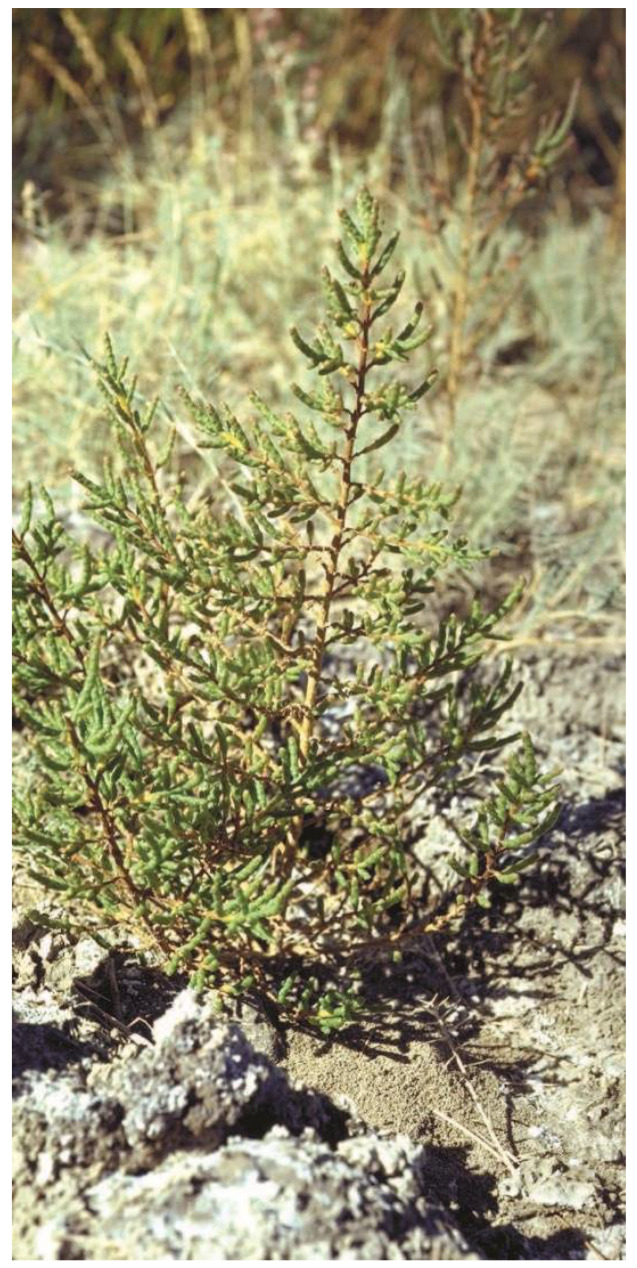
Photograph of *Salicornia herbacea* in its natural habitat (Finistère, France).

This flowering plant species belongs to the amaranth family and is called *S. herbacea* (Figure 3). This halophyte plant thrives in saltwater and can be referred to by the popular names dwarf glasswort and dwarf saltwort (e.g., salt marshes), having the capacity to collect more than 50% NaCl in dry weight [60]. It is indigenous to the coastal regions of China, southern and eastern USA, southern California, Belize and in coastal regions of Mexico (on both the west and east coastlines) [61,62,63]. *S. herbacea* is extremely promising as a domesticated oilseed, biomass and forage crop plant [3,64,65]. In light of all this, its evaluation as an oilseed and vegetable crop in the desert coasts of Mexico, Africa and the Middle East is favorable [41,44,48,66]. Seeds germinate immediately in seawater and possess a high percentage of protein (35%) and oil (30%) but very little salt (3%) [52]. Given the large amount of polyunsaturated linoleic (75%) and linolenic (omega 3) fatty acids, both quality and oil yield are equivalent to those presented by major oilseed crops. Oil can be utilized to make biodiesel and is valuable for human consumption [46,67,68]. Additionally, high-salt drainage water, e.g., the effluent that derives from farming in the Central Valley of California, can be used to irrigate plants [41]. Aquaculture farms in Eritrea’s wastewater have been utilized to produce the plant, which is then collected to be used as animal feed [64,67].

According to studies by Lu et al. [69], *Salicornia* plants are an excellent source of a number of vitamins, dietary fiber, the 18 essential amino acids, unsaturated fatty acids and microelements such as calcium, iodine iron and zinc. The plant may also provide raw materials for pharmaceutical chemical manufacturing purposes and biological salt [70].

## 6. Commercial Potentials towards Application in Various Industries

**Figure 4:**
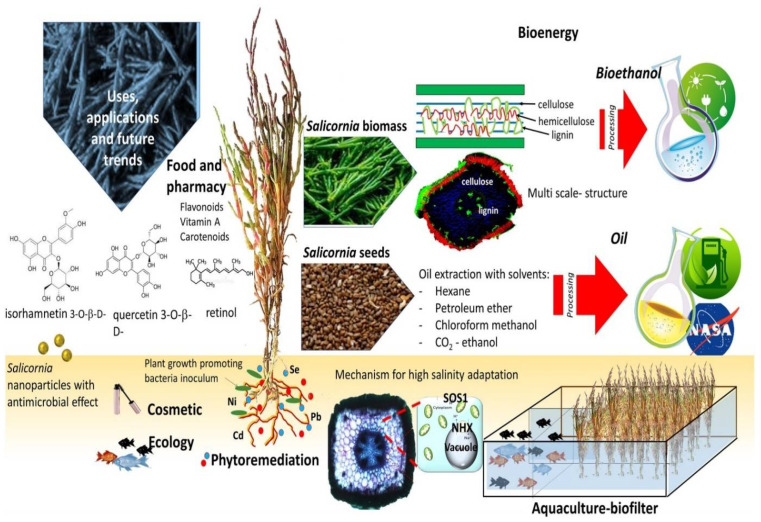
A summary of potential *Salicornia* applications [73].

Interest is being shown in the potential applications of *Salicornia*, its uses and future trends (see Figure 4). Apart from it being utilized on the food market for human consumption, the plant can be used as a biofilter for mariculture effluents, be converted into bioethanol and employed as aviation biofuel [71,72,73].

Due to *Salicornia* plants’ contents of flavonoids, vitamin A, carotenoid, retinol and quercetin, they have many applications in the food and pharmacy markets [73]. *Salicornia* seeds are valuable because 30% of edible oil can be obtained from them, and the remainder can be utilized as fodder. There is a very high economic potential value in *Salicornia* seeds because they are an excellent source of polyunsaturated oil. Extracted oil from *Salicornia* seeds have been utilized as biofuel by the National Aeronautics and Space Administration (NASA) researchers in space expeditions [73]. According to previous research, approximately eight lipid patterns of *Salicornia* oil have been obtained from its seeds [74,75]. Kang et al. [76] and Min et al. [77] report that *S. herbacea* leaves and seeds are employed for their antioxidant properties. Additional details on the utilization of Salicornia as an ingredient in functional foods is provided under Section 8.2 “*Utilization as functional foods*”. Ahmed and co-workers reported on the synthesis of gold nanoparticles using *S. brachiata*, which had antibacterial and catalytic activity, with great potential applications in the food, pharmacy and cosmetic industries [78].

Apart from the commercial potential in the food industry, the pharmaceutical, cosmetic and aquaculture industries also benefit from the utilization of *Salicornia*. In addition, phytoremediation of saline soils polluted with heavy metals promotes its ecological role. In particular, bio-saline farming offers a feasible solution in marginal, coastal and salt-affected areas to utilize unconventional water resources such as seawater, brackish groundwater and the reject brine from desalination [79]. Much interest has been shown in living soil bacteria with proven beneficial effects on plants. For example, utilizing plant-growth-promoting rhizobacteria as bio-inoculants for enhancing the growth of several halophytic plants such as *Salicornia* has been described in the works of El-Terabily et al.; Mesa-Marín et al.; and Jímenez-Mejía et al. [80,81,82]. Actinobacteria *Streptomyces euryhalinus* and *Actinoplanes deccanensis* have been isolated from marine environments to promote the growth and seed yields of *S. bigelovii* by stimulating endogenous levels of polyamines and other plant growth regulators [83].

The opportunities to extend the *Salicornia* plant market can be further expanded when plant yields increase, which includes its roots, shoots and seeds. Mathew et al. have reported that individually treating *S. bigelovii* in vitro with some *S. bigelovii* rhizosphere actinobacteria, i.e., *Streptomyces chartreusis*, S*. tritolerans* and *S. rochei,* respectively, brings about 46.1%, 60.0%, and 69.1% increases in seed yields [84]. However, synergetically combining these three strains leads to increases in dry shoots and root biomass of 62.2% and 77.9%, respectively, while seed yields grow by 79.7%. Similarly, El-Tarabily et al. report that actinobacteria *Actinoplanes deccanensis* United Arab Emirates (UAE1) and *Streptomyces euryhalinus* UAE1, with very high polyamine production, have a synergistic effect by increasing the length and dry weight of *S. bigelovii* shoots (by 44.6% and 26.11%, respectively), roots (by 42.3% and 26.5%, respectively) and photosynthetic pigment and seed yields (by 57% and 41.4%, respectively) compared with the control plants left under greenhouse conditions [83].

The unique ability of *Salicornia* to adapt to salt is based on the reduction in the toxicity of sodium ion in the cytoplasm, restraining the vacuoles in order to maintain a normal cellular turgor pressure [34,73]. The signal transduction pathways of the plant have a vital role for the connection within the mechanism with regard to sensing and genetic response [85]. High levels of salinity interfere with plant growth since they lead to ion toxicity and physiological drought. Gene expression regulation and changes in transcript levels are part of plant salt-stress adaptation [73].

The first mechanism by *Salicornia* to overcome high Na^+^ concentrations is the water storage in the parenchyma. This will dilute the accumulated salts, thus helping to maintain cellular turgor, allowing the plant to cope efficiently with high salinity [86].

According to Yadav and co-workers, *Salicornia species* can use three different techniques for preventing and adapting to high salt concentrations: (1) active sodium efflux, (2) sodium compartmentalization in vacuoles and (3) inhibition of sodium, where antiporters (group of genes), such as the Salt Overly Sensitive 1 (SOS1) gene that encodes a plasma membrane Na^+^/H^+^ antiporter, have a regulating role in ion homeostasis of plants [87].

## 7. Techniques to Produce *Salicornia* as a Salt Substitute

In order to utilize *Salicornia* as a salt substitute, the first step involves collecting the plant. This is followed by a cleaning procedure, which slightly differs according to reports by several authors [16,88,89,90]. Plants may be washed with either seawater or salty water [90]. Then, they are normally cut and heated [16,79], or may be dried as reported by Ghosh et al. [88]. Drying is an important process that permits plants to be stored for a longer time, such as freeze-drying and oven-drying [89]. However, the drying procedure must be carefully carried out to avoid damaging plants [91]. After these steps, different methods may be included, some of which may incorporate filtration [16,88] or mix the extract with distilled water [21,88].

A standard procedure involves cleaning the fresh stalks of *S. bigelovii* under tap water, washed at 95–98 °C for 4–6 min, quickly cooled with water and broken down in a high-speed tamping machine homogenizer (DS-1, Yoycart, Shanghai, China). After filtering with 200 mesh gauze, the filtrate is concentrated by rotary evaporators at 85 °C (vacuum degree at 0.06 MPa) to give liquid plant salt, which is stored at −20 °C to prepare plant salt feed [92].

In another procedure, when *S. bigelovii* is harvested fresh, it can be fractionated by using a twin-screw press (Angelia 8500S Angel juicer, Angel Co., Ltd., Busan, South Korea) equipped with a coarse screen with hole sizes of 1 mm [93]. The fiber produced is rewetted in a 1:1 weight ratio of fiber and saline irrigation water after the first fractionation and is pressed in the screw press for a second time. The resulting samples of juice and fiber are frozen, and the remaining biomass is dried at 70 °C and ball milled to a powder [93].

Other drying procedures reported by various authors for *S. bigelovii, S. brachiata* and *S. herbacea* include hydrothermal liquefaction as an efficient technology to convert high moisture content feedstock such as *S. bigelovii* stems to biofuel intermediates [94]. According to the authors, the process reduces process energy consumption due to the omission of the drying step and using water in the biomass as a potent reaction medium at elevated temperatures (180–375 °C) and pressures (4–30 MPa) [94]. Oven-drying of *S. brachiata* by oven-drying at 80 °C for 7 days [95]. Oven-drying of *S. herbacea* at 70 °C for 48 h [96].

The entire saltwort (*S. herbacea*) plant can be micronized to develop a table salt substitute. In line with the Chinese National Standard GB/T 12457-90, the indirect precipitation titration method can be applied to establish the NaCl content of liquid plant salt [97]. Employing the entire plant can cut waste and provide beneficial effects compared with plant extracts. The micronization process can also cut waste and allow entire plants to be employed as food additives for preparing several health foods. Thus, applying micronization to entire plant sources has been paid plenty of attention and resulted in new food industry applications [92,98].

It is necessary to improve the characteristics of the micronized powders obtained from entire plant sources because their handling often tends to be difficult and their product flowability is low. One improvement can be made by employing fluid-bed dryers. The products acquired from fluid-bed coaters present excellent preservation characteristics, along with better flowability and controlled release, and they are more conveniently handled.

So, to develop saltwort as a table salt substitute, the entire saltwort plant is micronized with a pulverizer. The micronized powder is mixed with distilled water and converted into spherical granules by means of a fluid-bed coater. To enhance this powder’s dispersibility, the micronized powder is mixed with a solution that contains several soluble solid saltwort aqueous extract contents, which is made into spherical granules [21].

Instead of heating or drying plants, extraction from plants can be carried out with water after cutting. If extraction is performed at higher temperatures, sodium content increases, while amino acids decrease. The obtained result is inversely proportional when carried out at lower temperatures or for times shorter than 4–10 h [91]. This process can increase amino acids by up to 200–400% and lower sodium content to about 1–3%. Finally, the resulting product is then centrifuged and ultrafiltrated. The product can be utilized as salt substitutes made from *Salicornia* species plants [99,100]. Additionally, herbal salt with the oil of plants can be made by drying, incineration and filtration [99]. They are dried and pulverized to obtain a granulose extract [101].

## 8. Nutrition Profile and Health Impacts

One good option is to introduce *Salicornia* as a salt substitute for human consumption given its unique nutritional profile, as reviewed by several authors [21,99,102]. One research work into the mechanism and effect of *S. bigelovii* plant salt (SPS) on Sprague Dawley (SD) rats’ blood pressure revealed that edible salt induces hypertension, while SPS does not [24]. SPS as a salt substitute has a protective effect on the liver and kidneys and can improve the body’s antioxidant ability to protect the liver and kidneys from high salt intake damage and prevent hypertension [24].

The common names, geographical location, some highlights on the nutritional features and compositions including health benefits that are associated with the three species of interest included in this review article are shown in Table 1 below.

In the study by Ventura and co-workers (Table 1), it was shown that the *S. bigelovii* species has a high content of β-carotene (15.9 mg/100 g of fresh weight), as well as polyphenols at (1.2 gallic acid equivalent GAE/g) of fresh weight [107]. Kang et al. [103] stated that the seawater cultivation of *S. herbacea* could result in higher phenolic and flavonoid contents.

*S. bigelovii* is referred to as ‘green food’. It is apt for being developed into new plant salt as a salt substitute for preventing high blood pressure. Natural plant salt has been confirmed to be effective in controlling blood pressure and reducing morbidity from cardiovascular and cerebrovascular diseases, i.e., coronary disease, cerebral apoplexy, etc. [103]. Nevertheless, no key studies into the relation linking *S. bigelovii* Torr plant salt (SPS), SPS intake and high blood pressure have yet been reported [24]. In this study, *Salicornia bigelovii* Torr. stalks were made into plant salt for investigating the impact of a high-dose intake of SPS on the blood pressure of SD rats. The results will offer a sound basis for employing SPS as substitute salt to prevent and control high blood pressure [24].

Min et al. [77] reported the chemical composition and micronutrients from different parts of the *S. herbacea* plant such as leaves, stems and roots. They concluded that the stems and roots of the plant contained a significant content of important amino acids such as aspartic acid (140.1 and 165.5 mg/100 g), glutamic acid (160.5 and 182.3 mg/100 g) and isoleucine (107.5 and 94.7 mg/100 g), respectively. In addition, they found a significant amount of minerals such as sodium (1218.1 mg/100 g), calcium (158.8 mg/100 g), potassium (740.1 mg/100 g) and magnesium (52.2 mg/100 g), mainly in stems. Other studies by Man and co-workers stated that the leaves of *S. herbacea* have significant contents of fatty acids such as linoleic and oleic. They also showed that *S. herbacea* contained bioactive compounds such as tungtungmadic acid and quercetin 3−0 glucoside [35].

*S. herbacea* consumption is considered to be a slight risk due to its iodine content, which can lead to hypokalemic [7]. It is noteworthy that, to date, no problem has been reported from consuming it. However, there has been some incidence of this hypokalemic rare disease being referred to as “throtoxic periodic analysis” (TPP), which has been associated with hyperthyroidism. As a result of this, there have been recommendations about being careful with iatrogenic iodine compounds, especially when consuming *S. herbacea* [108].

*S. herbacea* are also unique for having a salty taste and a smaller quantity of sodium, as well as their nutritional quality of fatty acid content [4,46,109,110], especially polyunsaturated [111,112], in addition to minerals [4,16,20,53,103,105,108,113,114], vitamins [4,53] and antioxidants [4,7,20,29,105], which enrich their nutritional profile [4] and make *Salicornia species S. herbacea*, *S. bigelovii* and *S. brachiata* good options for making salt substitutes.

*S. herbacea’s* efficacy against inflammation, oxidative stress, asthma, diabetes, hepatitis, gastroenteritis and cancer has been reported [105]. *S. herbacea* leaves and seeds can also be employed for their antioxidant properties, as, respectively, reported by Min et al. [77] and Kang et al. [103]. The results obtained with the present study indicate that no experimental group (fresh mullet (FM) and salted semidried mullet (SSDM)) had *Staphylococcus aureus*. This indicates that the semidried salted method followed by using natural salt and *S. herbacea* treatment for preservation purpose is capable of preventing microbial contamination and prolonging shelf life [104].

*Salicornia* seeds are valuable for the amount of edible oil (30%) that can be extracted from them, whereas the remainder is employed as fodder. Some studies report the presence of proteins, lipids, bioactive polysaccharides, dietary fibers, sterols, minerals (Ca, Fe, K and Mg) and flavonoids in *S. herbacea* [105]. One study has shown *S. herbacea* seed oil as being stable to oxidation, which renders its use as eligible in food processing [115]. Oil is composed of oleic acid, linoleic acid, palmitic acid, arachidic acidtocopherol (α, γ, δ type), chlorophyll, phenol and β carotene, which all confer it a long shelf life as evidenced by the study of Choi et al. [115]. These authors observed that oil remained rancidity-free over a 60-day dark storage period. 24-ethyl-δ(22)-coprostenol, stigmastanol and some other bioactive fatty alcohols have been detected in oil [111].

*S. bigelovii* salt has been found to prevent the hypertensive effects usually related to sodium chloride. In a study by Zhang et al., a lower serum creatinine level was observed with *S. bigelovii* consumption due to its ameliorative effect on the liver and kidneys [24]. The same authors also showed that Na(+)-K(+)-ATPase activity and superoxide dismutase (SOD) increased, while malondialdehyde (MDA) content lowered. This finding advocates a beneficial effect on the body’s antioxidant profile [24]. *S. bigelovii* species are particularly noteworthy for having high contents of both β-carotene (15.9 mg 100 g^−1^ fresh weight-FW) and polyphenols (1.2 GAE g^−1^ FW) [107]. Kang et al. [103] report that *Salicornia* seawater cultivation may result in high flavonoid and phenolic contents.

In *S. brachiata*, a high content of sulfur-rich amino acids (cysteine and methionine) was detected because of the disruption of the sulfur bonds and release of these amino acids under stress [116]. Eganathan and co-workers identified the fatty acids present in *S. brachiata* fat as palmitic (16.48%), myristic (12.88%), oleic (32.79%) and, in particular, 10-undecenoic (37.85%), which has the potential for commercial exploitation for use in lubricants or pheromones in crop pests [109].

The metabolomic analysis of *S. brachiata* detected rich polyunsaturated fatty acids (PUFAs) and sulfur amino acids at up to 55–64% [116]. The authors also detected that selenium was present in *S. brachiata* [116]. Selenium is a micronutrient that is vital for growth with marked antioxidant effects whose deficiency can impair the immune system [63]. This justifies evaluating selenium extraction from *Salicornia* for human diet. Another study also revealed that *S. brachiata* is capable of absorbing nickel, arsenic and cadmium salts [117], which indicates that *Salicornia* plants can be an excellent source of the phytoremediation of heavy-metal-polluted saline coastal regions.

### 8.1. Antioxidant and Anti-Inflammatory Effects

The health impacts of *Salicornia* spp. are enhanced by its important role as an antioxidant and anti-inflammatory when added as a food ingredient. Plant-derived antioxidants are extracted from natural plants such as *Salicornia* spp. with antioxidant properties, they can effectively inhibit or delay the oxidative decomposition and deterioration of food ingredients, thereby enhancing the stability of food and extending their storage period [118].

*Salicornia* can be utilized as ‘functional foods’ since it contains metabolites with bioactivities [118]. For example, the *S. brachiata* plant possesses nutritional antioxidants, scavenging activities, amino acids, flavonoids, essential fatty acids and polyunsaturated fatty acids, making it a promising ideal plant that can used as a functional food or as a dietary supplement in nutraceutical industries [116]. An interesting application that uses *S. brachiata* to produce low-cost antibacterial nanoparticles for the food and pharmacy markets was created by Ahmed et al. [78].

In previous research, it was demonstrated that *S. bigelovii* contains a variety of chemical components with antioxidant properties [119], antitumor properties [119] and the molecular basis for anti-inflammatory effects [120]. The effect of *S. bigelovii* extract on the preservation of aquatic products as demonstrated by Wang and co-workers showed that *S. bigelovii* can significantly reduce the pH value and the content of total volatile basic nitrogen (TVB-N) in fish meat, leading to the reduction in amines by inhibiting the decomposition of protein and the oxidation of unsaturated fatty acids [118]. The antioxidant compound that was purified from *S. herbacea*, known as a 13-Oxo-9(Z), 11(E)-octadecadienoic acid, i.e., ‘13-KODE’, produced anti-inflammatory effects with great potential for use in treating inflammatory diseases [121].

Several natural compounds have been isolated from *S. herbacea*. Methanol-extracted compounds from *S. herbacea* includes stigmasterol, P-sitosterol, uracil and isorhamnetin-3-0-β-D-glucopyranoside [111]. The presence of several chlorogenic acid derivatives, such as 3-caffeoyl-4-dihydrocaffeoyl quinic acid, also known as tungtungmadic acid [122], methyl4-caffeoyl-3-dihydrocaffeoyl quinate (salicornate), methyl 3,5-dicaffeoyl quinate, 3,5-dicaffeoylquinic acid and 3,4-dicaffeoylquinic acid, as well as the flavonoid derivatives quercetin 3-0-p-o-glucopyranoside and isorhamnetin 3-0-p-o-glucopyranoside and isoquercetin 6″-0-methyloxalate, were also obtained [123].

Other authors have reported the isolation of isorhamnetin 3-0-p-o-glucopyranoside and quercetin 3-0-p-o-glucopyranoside from the n-butanol extract of *S. herbacea* [124,125,126].

In 2007, Oh et al., obtained *S. herbacea* viscozyme-treated extract and further extracted it with ethanol. This allowed the isolation of simple phenolic acids such as protocatechuic, caffeic and ferulic and also the flavanol derivatives quercetin and isorhamnetin [127]. Chung and co-workers also found that tungtungmadic acid prevents iron-induced liver microsomal lipid peroxidation (IC50 9.3 μM) and is effective in protection of plasmid DNA against strand breakage induced by hydroxyl radicals [122]. Hwang and co-workers reported that tungtungmadic acid is capable of inhibiting tumor cell invasion and migration in human fibrosarcoma HT-1080 cells by regulating protein kinase co-dependent matrix metalloproteinase-9 expression. They proposed that the anti-invasive effects occur through the inhibition of activator protein-1 (AP-1) and signaling pathways, which involves protein kinase C-delta (PKCծ) and three mitogen-activated protein kinases, i.e. extracellular signal-regulated kinases (ERK), a subfamily of mitogen-activated protein kinases that are responsive to stress stimuli (p38MAPK) and terminal kinases (JNK-c-Jun N) that belongs to the mitogen-activated protein kinase family leading to the down regulation of matrix metallopeptidase 9 (MMP-9)—key effectors of extracellular matrix remodeling that play a role in inflammation [128].

### 8.2. Utilization as Functional Foods

The utilization of these *Salicornia* species as functional foods lies in their compositional quality attributes and health impacts discussed above. For example, the chemical composition of *S. bigelovii* seeds had protein (31.2), ash (5.5), fiber (5.3) and oil (28.2), with a good balance of fatty acids (linoleic 74, linolenic 2.6, oleic, 2.5 palmitic 8.1 and stearic 2.2) [29]. The seeds from these *Salicornia* species are considered very valuable, since 30 edible oils can be extracted from them, and the rest of the biomass can be used as fodder. The seeds and leaves of *S. herbacea* are used due to their antioxidant properties, as reported by [76,77]. The presence of polyunsaturated oil in *Salicornia* seeds makes them of high economic value.

When monosaccharide composition in *S. brachiata* was analyzed, the obtained fractions revealed that mannose, rhamnose, galactose, arabinose and glucose dominated, with a meager presence of xylose and ribose [129]. The MALDI-TOF (matrix-assisted laser desorption/ionization time of flight mass spectrometry) proteomic analysis demonstrated that *S. brachiata* seeds contained a considerable amount of protein. In the globulin proteins of *S. brachiata*, high disulfide linkages were detected, and sulfur-rich proteins were noted to be suitable for nutritional purposes, which makes a strong case to consume it [54].

*S. herbecea* enhanced fermenting microbe propagation and improved the quality of vinegar quality [130]. The authors noted that *S. herbecea* activates the growth of microorganisms during the fermentation of nuruk, makgeolli, or vinegar but also serves as a nutritional supplement that improves the quality of vinegar [130]. The young stalks of *S. herbacea*, called hamcho and tungtungmadi in Korea, are consumed in a variety of ways such as a seasoned vegetable, salad and fermented food in coastal areas of Korea; they are also utilized as a main ingredient of salad in Europe [111,131]. In India, shoots of *S. herbacea* can also be transformed into beverages such as nuruk (fermentation starter), makgeolli (Korean rice wine), or vinegar [130]. Kang and co-workers reported that the seeds of *S. herbecea* are also used as tea [76].

The annual glasswort or saltwort *S. bigelovii* Torr. are used freshly in salad or boiled for jarring as pickles with vinegar, sugar, onion, bayberry leaves and mixed pickling spice in Nova Scotia, Canada [111,132]. The aerial parts of *S. bigelovii* have also been used as an ingredient in vinegar in Italy and France [111,123]. *S. bigelovii* is described as a very valuable source of minerals, amino acids and antioxidants that render it the most promising salt-loving plant for food use [49].

In the efforts to create a halophyte-based food industry with local produce in a desert environment, a project funded by Universal Exposition in Dubai (EXPO2020 Dubai) from 2019–2021 entitled ‘From Desert Farm to Fork: Value chain development for innovative halophyte-based food products’ will utilize the health-giving properties in *S. bigelovii* in newly formulated food products [79].

The following food products were developed with *S. bigelovii*: sorbet with mango, banana and *S. bigelovii*; camel laban with *Salicornia*; lasagna with *Salicornia*; charcoal bread with vegan *Salicornia* burger; falafel with *Salicornia*, quinoa, chickpea and kale; vegan *Salicornia* burger; steamed *Salicornia* bread; camel cheesecake with *Salicornia*; charcoal pizza with *Salicornia*; and vegan *Salicornia*, quinoa and peas balls. Such initiatives can be replicated industrially in similar climatic contexts and salt-affected areas. 

The ratio of *S. bigelovii* as an ingredient in the recipes ranged between 20% and 40% for all the products and recipes that were developed. In this project, it was observed that *Salicornia* showed great versatility in cooking options and processing possibilities for both salty and sweet dishes, liquid and solid food products. As already discussed in the previous section, *Salicornia* is characterized by its antioxidant and anti-inflammatory effects, a high content of minerals and high vitamin C, especially at a later growth stage, which when combined with its rich content in zinc, magnesium and manganese will make it a good candidate to boost the immune system [79].

## 9. Conclusions and Future Perspectives

The role of *Salicornia* as a good salt substitute is very promising. It is quite sustainable as an underutilized flora and can form part of the human diet. It can also provide a source of income for growers. The three species considered in this review, namely *S. bigelovii, S. brachiata* and *S. herbacea,* are rich in dietary bioactive components, low in sodium and high in protein and fiber and can promote health. The salt-water-tolerant plant also helps to improve water quality through remediation and makes clean water available that is essential for food processing and sanitation. There is a need to investigate the nanoparticles with antibacteria activities that are found in *S. brachiata*; they will be useful in the food, pharmacy and cosmetic industries.

However, one major drawback of utilizing *Salicornia* is its non-perennial nature. So, it cannot be harvested all year round, unlike its counterpart genus *Sarcocornia,* which is non-seasonal and can be supplied all year long. For example, the native range of *S. bigelovii* plant is coastal salt marshes, where it germinates in winter or spring and flowers in summer in response to photoperiod [133]. The plants senesce and shed seeds in the fall. In order to overcome this challenge, efforts are being made to ensure the regular availability of *Salicornia* plants with efficient drying techniques in the future.

## Figures and Tables

**Table 1:** The common geographical location of the three *Salicornia* species and their health benefits. Adapted from ref. [7].

*Salicornia* Species	Common Name	Location	Nutritional Features and Compositions	Health Benefits	References
*S. bigelovii*	Dwarf saltwort	USA, Mexico	β-carotene (15.9 mg/100 g). polyphenols (1.2 GAE/g) Na (30.4 g/kg), Cl (45.8 g/kg), K (13.2 g/kg)	protective effects on cardiovascular diseases, hypertension	[24,103]
*S. herbacea*	Dwarf glasswort	South Korea	presence of tungtungmadic acid, quercetin	protective effects on diabetes, hepatitis, gastro-enteritis	[20,76,104,105]
*S. brachiata*	Umari keerai	India	high cysteine and methionine	antioxidant effects, immune booster	[54,106]

## Data Availability

Not applicable.
